# Quantitative assessment of liver steatosis using ultrasound controlled attenuation parameter (Echosens)

**DOI:** 10.1007/s10396-021-01106-1

**Published:** 2021-06-16

**Authors:** Giovanna Ferraioli

**Affiliations:** Department of Clinical, Surgical, Diagnostic and Pediatric Sciences, Medical School University of Pavia, Viale Brambilla 74, 27100 Pavia, Italy

**Keywords:** Controlled attenuation parameter, Liver steatosis, Ultrasound, NAFLD, Attenuation coefficient

## Abstract

Controlled attenuation parameter (CAP) is the algorithm available on the FibroScan system (Echosens, France) for quantification of liver steatosis. It assesses the ultrasound beam attenuation, which is directly related to liver fat content. The inter-observer reproducibility of the technique is high, with a reported concordance correlation coefficient of 0.82. Specific quality criteria for CAP measurements are not clearly defined yet, and there are conflicting results in the literature. Using liver biopsy as the reference standard, several studies have assessed the CAP performance in grading liver steatosis, and have reported that values are not affected by liver fibrosis. The cutoff for detection of liver steatosis reported in the literature ranges from 222 decibels per meter (dB/m) in a cohort of patients with chronic hepatitis C to 294 dB/m in a meta-analysis of nonalcoholic fatty liver disease (NAFLD) patients. CAP has been used as a tool to noninvasively evaluate the prevalence of NAFLD in groups at risk or in the general population; however, it should be underscored that different CAP cutoffs for steatosis detection (S > 0) were used in different studies, and this limits the robustness of the findings. CAP, alone or combined with other noninvasive indices or biomarkers, has been proposed as a tool for assessing nonalcoholic steatohepatitis or as a noninvasive predictor of prognosis in patients with chronic liver disease. CAP is easy to perform and has become a point-of-care technique. However, there is a large overlap of values between consecutive grades of liver steatosis, and cutoffs are not clearly defined.

## Introduction

Currently, with the availability of highly effective all-oral direct-acting antivirals for the eradication of hepatitis C virus (HCV), nonalcoholic fatty liver disease (NAFLD) has become the most common cause of chronic liver disease, due to an increasing rate of obesity and to a sedentary lifestyle. Recently, a panel of experts proposed the adoption of a new term based on a holistic approach to the disease, i.e., metabolic dysfunction-associated fatty liver disease (MAFLD) [1, 2]. The diagnosis of MAFLD is based on the evidence of liver steatosis together with three positive criteria: overweight/obesity, presence of type 2 diabetes mellitus, or evidence of metabolic dysregulation [1].

The prevalence of NAFLD in the general population is around 25%, and the disease has a large spectrum, ranging from benign steatosis to a more severe condition that may lead to cirrhosis with its complications [3]. In this setting, the availability of tools able to noninvasively quantify the amount of fat in the liver is highly desirable.

Controlled attenuation parameter (CAP) is the tool available on the FibroScan system (Echosens, Paris, France) for the quantification of liver steatosis. It assesses the attenuation of the ultrasound (US) beam, which is directly related to liver fat content. The result is expressed in decibels per meter (dB/m) and ranges from 100 to 400 dB/m; it is given together with the liver stiffness value.

The technique is available on the M and XL probe of the FibroScan system, and the choice between the two probes is based on the skin-to-liver capsule distance (up to 25 mm or higher than 25 mm). Based on this parameter, the system software automatically suggests the choice of the probe.

It is worth mentioning that a correct choice of the probe is of outmost importance. In fact, a study performed in a cohort of patients using liver biopsy as the reference standard has reported that the use of the M probe in patients with a skin-to-liver capsule distance higher than 25 mm leads to an overestimation of liver steatosis [4]. The rate of failure, which was initially reported at 7.7% of cases in a large prospective study including 5323 examinations [5], is around 3% when both probes are available [6, 7].

This review analyzes the current literature regarding the use of CAP, providing an overview of its applications and discussing the limitations of the technique.

## Reproducibility and quality criteria

Using the M probe, a study on 351 individuals (104 consecutive patients and 247 healthy volunteers) reported that the inter-observer reproducibility of the measurements was high, with a concordance correlation coefficient of 0.82, even though the agreement between observers decreased to 0.65 for body mass index (BMI) ≥ 30 kg/m^2^ and to 0.44 for CAP values < 240 dB/m [8]. The lower inter-observer agreement between measurements in obese subjects can be explained by the use of the M probe, which was the only one available at the time of the study. The fair agreement in subjects who likely had no steatosis might be due to the uneven distribution of other tissue scatterers that prevail over fat content in determining the attenuation of the US beam.

Another study, in which both the M and the XL probe were used, also found high intra-observer and inter-observer correlations (*r* = 0.82 and *r* = 0.70, respectively). However, the intra-observer correlation decreased with the XL probe (M probe vs. XL probe: *r* = 0.85 and *r* = 0.75, respectively) [7].

Generally, investigators have used the quality criteria that were set for liver stiffness measurements, namely median value of 10 acquisitions with an interquartile range/median (IQR/M) up to 30%. Using these criteria, every CAP measurement that is obtained together with a reliable liver stiffness measurement is accepted as reliable.

Specific quality criteria for CAP measurements are not clearly defined yet, and there are conflicting results in the literature [6, 9–11]. In a multicenter study including 754 patients with mixed etiologies of liver disease who were studied with the M probe before liver biopsy, it was shown that the CAP accuracy significantly decreased when the IQR of the 10 consecutive CAP acquisitions was higher than 40 dB/m [9]. Another study on 119 NAFLD individuals, who underwent magnetic resonance imaging-derived proton density fat fraction (MRI-PDFF) as the reference standard for the quantification of liver steatosis, reported that the IQR value should less than 30 dB/m [10]. However, it is important to note that the use of these IQR values as quality criteria was not confirmed in a recent multicenter study that included 380 NAFLD subjects who underwent liver biopsy [6]. Likewise, a recent meta-analysis that included individual data from 2346 patients studied with the XL probe also demonstrated that the use of quality criteria, either IQR < 40 dB/m or IQR/M at different thresholds (≤ 30%, ≤ 20%, or ≤ 10%), did not improve the performance of CAP in grading liver steatosis [11].

## Accuracy and cutoff values

Using liver biopsy as the reference standard, several studies have assessed the performance of the CAP in grading liver steatosis [11–17].

Studies that were performed using liver histology as the reference standard reported that the CAP values were not affected by liver fibrosis and cirrhosis [13, 14, 18]. The cutoff for the detection of liver steatosis reported in the literature ranges from 222 dB/m in a cohort of patients with chronic hepatitis C [13] to 294 dB/m in a meta-analysis of NAFLD patients [11].

Of note, studies that were performed in patients with chronic viral hepatitis reported cutoffs for grading liver steatosis lower than those obtained in cohorts of patients with mixed etiologies of liver disease or NAFLD. Therefore, it has been suggested that the cutoffs are etiology-specific. However, it must be highlighted that it is likely that the disease prevalence in the studied population rather than the etiology of liver disease accounted for this difference [19]. In fact, the CAP quantifies the attenuation of the US beam, which is directly related to the liver fat content that has the same histological appearance no matter what the etiology of liver disease is. The only difference is between macrovesicular steatosis, i.e., the commonest type, and microvesicular steatosis. The latter is an uncommon and worse condition, observed in cases with an inherited or acquired defect in beta-oxidation of fatty acids [20].

In an individual patient data meta-analysis that included 19 studies in 2,735 patients with mixed etiologies of liver disease (20% with NAFLD) studied with the M probe, the optimal cutoff values were 248 (237–261) dB/m for S > 0, 268 (257–284) dB/m for S > 1, and 280 (268–294) dB/m for S > 2 [17]. Of note, the cutoffs were affected by several covariates including NAFLD, diabetes, and BMI. Based on this finding, it has been proposed to add to the optimal cutoff values 10 dB/m in cases of NAFLD/NASH or diabetes, and subtracting/adding 4.4 dB/m for each unit of BMI below/above 25 kg/m^2^ in the BMI range 20–30 kg/m^2^. In this meta-analysis, the areas under the receiver-operating characteristics curves (AUROCs) for detecting and grading liver steatosis were higher than 0.80; however, for the detection of steatosis (S > 0), the sensitivity was suboptimal (69%).

A multicenter study, which included 383 NAFLD patients with valid FibroScan and biopsy results, reported that the CAP detected steatosis (S > 0) with an AUROC of 0.87, and the optimal cutoff was 302 dB/m. Of note, the difference between this cutoff and the one for S > 2 was very small (35 dB/m) [6].

It should be underscored that a recent individual patient data meta-analysis that included 13 published studies in 2346 patients who were assessed with the XL probe reported that the CAP was unable to satisfactorily grade steatosis in the 1277 patients with NAFLD [11]. The cutoff for detecting steatosis in NAFLD was 294 dB/m, with a difference in cutoff values between S > 0 and S > 2 of 37 dB/m. This figure was similar to the one observed in the study of Eddowes et al. [6] and to the mean absolute difference observed between the M probe and XL probe in this same meta-analysis (30 dB/m). On the other hand, uncertainty still remains about the cutoff value to be used in real-word clinical practice for steatosis detection (S > 0) [19].

Of note, in a series of patients with different causes of liver disease (chronic hepatitis B virus, chronic hepatitis C virus, and NAFLD), no significant difference in CAP values for individual steatosis grades was found across etiologies [14].

Using the imperfect gold standard methodology in a series of 726 subjects, CAP performed significantly better than US for assessing liver steatosis in patients with chronic viral hepatitis and advanced liver fibrosis [21]. Indeed, an increase in liver echogenicity can be observed in both advanced fibrosis and steatosis, and this may account for the decreased accuracy of conventional US in these patients.

Algorithms for quantifying the attenuation of the US beam are currently available on high-end US systems. ATI, which is the algorithm developed by Canon and implemented in the Aplio i800 (or higher) US systems, showed a significantly higher correlation with MRI-PDFF and was more accurate than CAP for detecting and grading steatosis [22]. This difference reached a statistical significance for S > 1 steatosis. The US-guided attenuation parameter on the GE systems also has shown a higher accuracy than CAP in a study that used liver histology as the reference standard [23].

An upgraded CAP algorithm called SmartExam is able to acquire much more data than the one currently available, and this may lead to an improvement in accuracy. Thus far, no study results that support this hypothesis have been published.

## Assessment of liver steatosis in children

There is an increasing incidence of NAFLD in children, mainly due to sedentary lifestyles and hyper-caloric diets that are the main drivers of the obesity epidemic. Even though benign steatosis is the most common type of NAFLD, the possibility of more serious conditions should not be overlooked. Indeed, it has been reported that 17% of overweight or obese children with an alanine aminotransferase (ALT) increase, who are referred from primary care to pediatric gastroenterology after screening, have advanced fibrosis (F3) at diagnosis [24]. Furthermore, the disease in children seems to be more severe than in adults [25]. Therefore, a timely diagnosis in this setting is highly desirable. B-mode US, which is the most widely used imaging technique for screening purposes, has a low sensitivity for the detection of small amounts of fat in the liver.

Unfortunately, the studies that have been carried out in children to evaluate the diagnostic performance of CAP are generally underpowered, including small numbers of subjects. As observed in adults, the cutoff value for the diagnosis of steatosis ranges from 225 dB/m in an unselected pediatric population [26] to 277 dB/m in a series of severely obese children [27]. This difference is likely due to the disease prevalence in the studied cohorts.

In a series of 69 children undergoing liver biopsy for standard clinical care, 23 had steatosis on biopsy. The optimal cutoff for S > 0 was 225 dB/m with 87% sensitivity, 83% specificity, and 0.93 AUROC [26]. Using MRI-PDFF as the reference standard, a study in 60 children with severe obesity found that the accuracy of CAP for detecting steatosis was higher than that of US, even though this difference did not reach statistical significance (AUROC: 0.80 vs. 0.68), probably due to the small number of children enrolled into the study [27]. In a study that included 86 children, 62% of whom were in the obese group, CAP showed 98.7% sensitivity and 80% specificity with an AUROC of 0.94 for diagnosing steatosis (S > 0) using a cutoff value of 241 dB/m, whereas the diagnostic performance for higher grades of fat content was suboptimal [28].

Using the imperfect gold standard methodology, a study in 305 children showed that both CAP (at a cutoff value of 249 dB/m) and the US score had a specificity above 90% for the diagnosis of steatosis, but the sensitivity of CAP was higher (72% vs. 46%) [29].

## CAP for assessing the prevalence of NAFLD

CAP has been used as a tool to noninvasively assess the prevalence of NAFLD in groups at risk or in the general population. It should be highlighted that different CAP cutoffs for the detection of steatosis (S > 0) were used in different studies, and this limits the robustness of the findings. Apart the uncertainty about the optimal cutoff of CAP for detection of steatosis, it is likely that an overestimation or underestimation of liver steatosis prevalence occurred in the published studies.

Using a CAP cutoff value of 274 dB/m, it was reported that the prevalence of NAFLD and MAFLD in the United States population was 37.1% and 39.1%, respectively, and it was higher among Hispanic individuals, whereas the lowest rate was observed among non-Hispanic blacks [30].

With a CAP of 248 dB/m or higher, 20.7% of young adults (around the age of 24 years) in the United Kingdom had steatosis, with 10% of them presenting severe steatosis as defined by a CAP value of 280 dB/m or higher [31]. Using the same CAP cutoff, the prevalence of NAFLD in a community-based study from a Mediterranean area was 48%, and 6.5% of people with NAFLD had vibration-controlled transient elastography (VCTE) values suggestive of advanced fibrosis [32]. Besides metabolic risk factors, PNPLA3 G variant and M6SF2 T variant were independently associated with NAFLD.

HIV-positive patients are at higher risk of NAFLD than the general population. Using a CAP cutoff of 248 dB/m in a series of 1511 mono-infected HIV patients (57.4% of whom were lean), it was demonstrated that the prevalence of NAFLD in HIV + lean patients was 24.2%, which is higher than the rate of 7–20% reported in the literature for lean subjects without HIV [33]. Moreover, lean NAFLD mono-infected HIV individuals had a higher prevalence of significant liver fibrosis than lean mono-infected HIV individuals without NAFLD (15.7% vs. 7.6%, respectively). However, it must be emphasized that sensitivity analysis performed in the same study with a CAP cutoff of 288 dB/m showed that the prevalence of lean NAFLD was only 4.4%, which is lower than that reported in the literature for lean subjects without HIV infection [33–35].

Using a CAP cutoff of 222 dB/m in a large Asian cohort of diabetic patients from primary care and hospital clinics, it was reported that around 70% had increased CAP values, and around 18% had an increase of liver stiffness [36]. Dyslipidemia, high BMI, increased ALT, fasting plasma glucose level, and non-insulin use were associated with increased CAP values.

Using a CAP cutoff value of 238 dB/m to identify steatosis in a series of 300 HIV mono-infected patients (90% on antiretrovirals), it was reported that NAFLD was present in 48% of cases, and it was independently associated with overweight/obesity and elevated ALT [37]. Of note, 15% of individuals in this series had significant fibrosis, as defined by a VCTE value of 7.1 kPa or higher, and the predictors were diabetes, elevated ALT, and use of protease inhibitors.

## Prognostic value of CAP

CAP, alone or combined with other noninvasive indices or biomarkers, has been proposed as a tool for assessing NASH or as a noninvasive predictor of prognosis in patients with chronic liver disease. In a multicenter study using liver biopsy as the reference, a score (FAST score) that combined liver stiffness measured by VCTE, CAP, and aspartate aminotransferase (AST) was derived. The FAST score cutoffs were 0.35 for ruling out and 0.67 for ruling in NASH, an elevated NAFLD activity score, and significant fibrosis. In the external validation cohorts, the positive predictive value ranged from 0.33 to 0.81, and the negative predictive value from 0.73 to 1.0 [38].

A study including 272 patients with compensated advanced chronic liver disease (cACLD) found that liver stiffness and CAP (XL probe) were independently associated with the risk of decompensation and severe bacterial infection, and a CAP value higher than 220 dB/m was associated with a decreased risk of decompensation (hazard ratio: 0.043) [39]. These findings are interesting, even though it must be highlighted that this was a retrospective study and only a small number of subjects experienced clinically relevant events in the follow-up period. Therefore, these results need to be confirmed in larger prospective studies. However, it is worth mentioning that the somehow “protective” effect of CAP values ≥ 220 dB/m for the development of the first occurrence of hepatic decompensation has also been reported in other published studies [40, 41]. A large study on 4282 individuals showed that increased CAP values were slightly protective for hepatic decompensations and hepatocellular carcinoma [41]. On the contrary, another study, in which only 15% of patients had NASH, showed that liver decompensation occurred significantly more frequently in cACLD patients with CAP ≥ 220 dB/m [42]. These conflicting results might be explained by differences in the study cohorts, mainly related to the etiology of liver disease. Of note, in several NAFLD/NASH cases with advanced liver disease, a small amount of fat in the liver was observed, and the term “burnt-out NASH” has been proposed [43].

## Monitoring changes in liver fat content over time

The usefulness of CAP for monitoring changes in liver fat content over time has been assessed in several studies, as reported below.

In a series of 507 NAFLD subjects who were followed up for a mean period of 21.2 ± 11.7 months, it was found that 16.5% of them had an increase in CAP value of at least 20%, while 39.6% had progression of liver stiffness of at least 20% [44]. In the subgroup of obese patients, which accounted for 45.8% of the whole cohort, the highest risk factors for CAP value increase were BMI and serum creatinine levels, whereas insulin resistance was an independent predictor of liver stiffness increase.

In a cohort of 326 HIV-infected patients, the increase in CAP values over a 1-year period was independently associated with an increase in BMI [45]. However, it must be highlighted that the increase in the CAP values reported in this study was not relevant. In fact, the median delta increase in CAP correlated with higher BMI was 14 dB/m.

In an Asian cohort of 611 patients with type 2 diabetes who underwent paired clinical assessments at baseline and at 3 years, the prevalence of NAFLD at baseline (as shown by a CAP ≥ 248 dB/m) was 67.6%. NAFLD developed in 52.0% of the 198 patients with a CAP < 248 dB/m at baseline, and in some 50% of these cases the increase was 280 dB/m or higher [46].

A study in 214 elderly Japanese patients with HCV genotype 1b who achieved sustained virologic response (SVR) after direct-acting antivirals (DAAs) treatment, found a significant increase in CAP values and total cholesterol from baseline to 48 weeks after the end of the treatment [47]. Similar results were observed in a series of 302 Japanese patients with chronic HCV genotype 2 infection treated with DAAs [48]. On the contrary, in a small cohort of 70 Japanese patients with chronic HCV infections and steatosis (as defined by CAP > 248 dB/m), CAP values decreased significantly, from 273 dB/m to 265 dB/m (*P* = 0.03) after HCV eradication with DAAs [49].

In an Italian multicentric cohort of 794 patients with HCV infection, the prevalence of steatosis (defined at a CAP value of 248 dB/m or higher) before starting DAA treatment was 46%. At 6 months after SVR, de novo steatosis developed in 29% of cases, whereas resolution was observed in 30% of cases, and both were associated with the presence of metabolic comorbidities [50].

It must be emphasized that, before using CAP as a tool to noninvasively monitor changes in liver fat content over time, it is important to define what is a meaningful change, i.e., change that is not the result of mere chance. In this regard, studies that have assessed the inter-observer concordance in the CAP measurements have shown that the mean difference in CAP values between two observers is up to 20 dB/m; therefore, this difference should be taken into account when following up patients [51].

## Is liver steatosis a confounding factor in fibrosis staging with shear wave elastography techniques?

CAP has been used as a tool to answer this question. A multicenter study that used liver biopsy as the reference standard demonstrated that the presence of severe steatosis, as assessed with CAP, led to an overestimation of liver fibrosis assessed by VCTE [52]. The risk of overestimation, namely false-positive results, was low for CAP values lower than 300 dB/m. The authors proposed an algorithm to identify the high risk of false-positive results in F2–F4 fibrosis and F3–F4 fibrosis, and recommended the use of NAFLD fibrosis score or liver biopsy in these cases [52]. Of note, all measurements were performed with the M probe; therefore, it is unclear whether the inclusion of the subcutaneous fat in some cases may have led per se to an overestimation of liver stiffness [53].

It must be underscored that the influence of steatosis on liver stiffness assessment was not confirmed in another study [6]. Likewise, the data from an individual patient data meta-analysis showed that the negative predictive value for ruling out significant fibrosis improved just slightly when the CAP values were taken into account; however, the CAP dependency was not significant [54].

## Conclusions

CAP is easy to perform and has become a point-of-care technique. However, it must be highlighted that there is a large overlap of values between consecutive grades of liver steatosis, and that the cutoffs are not clearly defined [55]. A recent meta-analysis questioned its value in grading liver steatosis in NAFLD patients [11]. When used to evaluate changes in liver steatosis over time, the minimum significant change must still be defined, taking into account that it must be higher than the difference observed in studies performed to assess the intra-observer and inter-observer variability. An upgraded algorithm has recently been released; however, no study aimed at evaluating whether this new version improves the performance is available yet in the literature.
